# Correlation of Renal Profiles with Choroidal Vascularity Index in Eyes with Diabetic Retinopathy

**DOI:** 10.3390/jcm10215155

**Published:** 2021-11-03

**Authors:** Jee Taek Kim, In Gul Lee

**Affiliations:** 1 Department of Ophthalmology, College of Medicine, Chung-Ang University, Seoul 06974, Korea; 2 Dangjin Bright Eye Center, Dangjin 31770, Korea; alkey@naver.com

**Keywords:** diabetic retinopathy, choroidal vascular index, subfoveal choroidal thickness

## Abstract

The vascular system is affected by systemic conditions, including diabetes, hypertension, and cardiovascular disease. The choroid is an important vascular tissue surrounding the sensory retina. However, the relationship between the choroid and systemic factors in patients with diabetes has rarely been investigated. Here, we assessed the correlation of renal profiles with choroidal vasculature in eyes with diabetic retinopathy (DR) using a retrospective study design. The study included 131 patients with diabetes who underwent swept-source optical coherence tomography and routine medical work-up within a 4-week period between 1 February 2016 and 30 October 2018. Consecutive patients with treatment-naïve DR who did not receive any subsequent treatment were included. The distribution of patients according to the DR stage was as follows: no DR, 32 patients; mild-to-moderate non-proliferative DR (NPDR), 33 patients; severe NPDR, 34 patients; and treatment-naïve proliferative DR, 32 patients. Multivariate regression analyses showed that the choroidal vascularity index (CVI) of naïve eyes with DR was negatively correlated with age (*p* = 0.013) and the serum levels of phosphorus (*p* = 0.002) and positively correlated with subfoveal choroidal thickness (*p* < 0.001). Taken together, our findings suggest that a lower CVI is associated with phosphorus retention in patients with chronic kidney disease.

## 1. Introduction

The choroid is an important vascular tissue surrounding the sensory retina and retinal pigment epithelium [1]. Diabetic retinopathy (DR) is one of the main causes of visual deterioration in the working-aged population in advanced and developing countries [2,3]. Changes in the choroid in patients with diabetes have been described in several studies using electron microscopic analysis, immunohistochemical analysis, ocular blood flow measurement, and indocyanine green angiography [4,5,6,7]. Since the detailed visualization of the choroid has become possible with the advent of enhanced-depth imaging optical coherence tomography (OCT), the choroid has been intensively investigated in eyes with DR. Moreover, with the advent of swept-source OCT, the better visualization of the choroid has become possible [8,9].

The choroidal vascularity index (CVI), which is the ratio of the choroidal luminal area to the total choroidal area, has been described as a novel marker that shows changes in several chorioretinal diseases [10,11,12,13]. Several studies have investigated changes in the CVI of eyes with DR. Recently, Tan et al. described a significantly decreased CVI in a patient with diabetes mellitus (DM) compared with that in controls [10]. More recently, Kim et al. and Gupta et al. also reported a significant decrease in CVI with worsening DR [14,15].

Diabetes is a chronic metabolic disorder and its complications manifest as the obliteration of small vessels and obstructions of large vessels in the body [16]. Vascular dysfunction eventually leads to tissue injury and degeneration [17]. Clinically, this causes various systemic complications, including cardiovascular disease, chronic kidney disease (CKD), and chorioretinopathy [18,19,20]. Recently, we investigated the correlation between subfoveal choroidal thickness (SFChT) and CKD in patients with DR [21,22]. However, CVI is known to be less variable [23]. We aimed to investigate whether the changes in the CVI of eyes with DR were correlated with CKD. Moreover, the relationship between the changes in CVI and kidney profiles in patients with diabetes has not been systematically investigated. Thus, the purpose of this study was to analyze the correlation of kidney profiles with CVI in diabetes patients with treatment-naïve DR eyes.

## 2. Methods

### 2.1. Ethics

This study was approved by the institutional review board committee of the Chung-Ang University Hospital, Seoul, South Korea, and adhered to the tenets of the Declaration of Helsinki. It was performed in a retrospective observational cross-sectional manner. The medical records and images of examinations of consecutive patients who visited both diabetes and retina clinics of Chung-Ang University Hospital between 1 February 2016 and 30 October 2018 were analyzed. Informed consent was waived by the approving IRB because of the retrospective nature of the study.

### 2.2. Inclusion and Exclusion Criteria

Only patients with treatment-naïve eyes who had not received any previous ocular treatment for DR or diabetic macular edema (DME) were included. A comprehensive ophthalmologic examination was performed, including the measurement of best-corrected visual acuity (BCVA), intraocular pressure (IOP), and refractive error. Slit-lamp examination, fundus examination and photography, and swept-source OCT (SS-OCT) were performed. For routine medical work-up, the following examinations were performed: blood pressure (BP) measurement; hemoglobin A1c (HbA1c) measurement; and the measurement of body weight, height, and body mass index (BMI). In order to check the detailed kidney function, the urine microalbumin and creatinine levels and the microalbumin/creatinine ratio of urine were analyzed. Only patients whose systemic work-up was performed within a span of 4 weeks from ophthalmic evaluations were selected.

Exclusion criteria included prior retinal surgery, focal laser, panretinal photocoagulation, intravitreal injection, or sub-Tenon injection; a history of ocular trauma; a history of any eye disease, including retinal and choroidal diseases; refractive error of more than ±3.0 diopters; and the presence of systemic diseases other than diabetes or hypertension. Eyes with low OCT image quality (image quality index < 90) or media opacities, such as dense cataract or vitreous hemorrhage, were also excluded.

Control data were obtained from the eyes of age-matched patients who visited the retina clinic for the treatment of idiopathic epiretinal membrane or macular hole.

### 2.3. DR Grading

DR severity was graded according to the Early Treatment Diabetic Retinopathy Study (ETDRS) retinopathy severity scale as follows: no DR, mild-to-moderate non-proliferative DR (NPDR), severe NPDR, and treatment-naïve proliferative DR (naïve PDR). Naïve PDR was defined as eyes with the neovascularization of the disc or retina or vitreous/preretinal hemorrhage. Severe NPDR was defined as any of the following: severe intraretinal hemorrhages in 4 quadrants; venous beading in 2+ quadrants; intraretinal microvascular anomalies in 1+ quadrants, and no sign of PDR. Mild-to-moderate NPDR was defined as the presence of microaneurysms only or more than just microaneurysms but less than severe NPDR. The diagnosis of DME was performed with fundus examination, according to the criteria reported by ETDRS, and confirmed on SS-OCT. In cases of severe NPDR or naïve PDR, fluorescein angiography was performed using an ultra-wide-field confocal scanning laser ophthalmoscope (Optos Panoramic 200MA™; Optos PLC, Dunfermline, Scotland, UK) to grade the severity of advanced DR.

### 2.4. OCT

SS-OCT was performed with a DRI Triton™ device (Topcon, Tokyo, Japan) at a wavelength of 1050 nm and a scan speed of 100,000 amplitude scans per second, which yielded an axial resolution of 8 μm and a depth of 2.4 dB/mm; OCT B-scan imaging was performed with a 6 × 6 mm 3D cube-scan and a 9 mm five-line cross-scan. Central retinal thickness (CRT) was obtained from the automatic ETDRS grid map in the 6 × 6 mm 3D cube-scan mode after the confirmation of the position of the grid. The SFChT was measured at the subfovea from the 9 mm five-line cross-scan using the built-in caliper tool as the distance between the Bruch’s membrane and the choroid–sclera interface.

### 2.5. CVI

**Figure 1:**
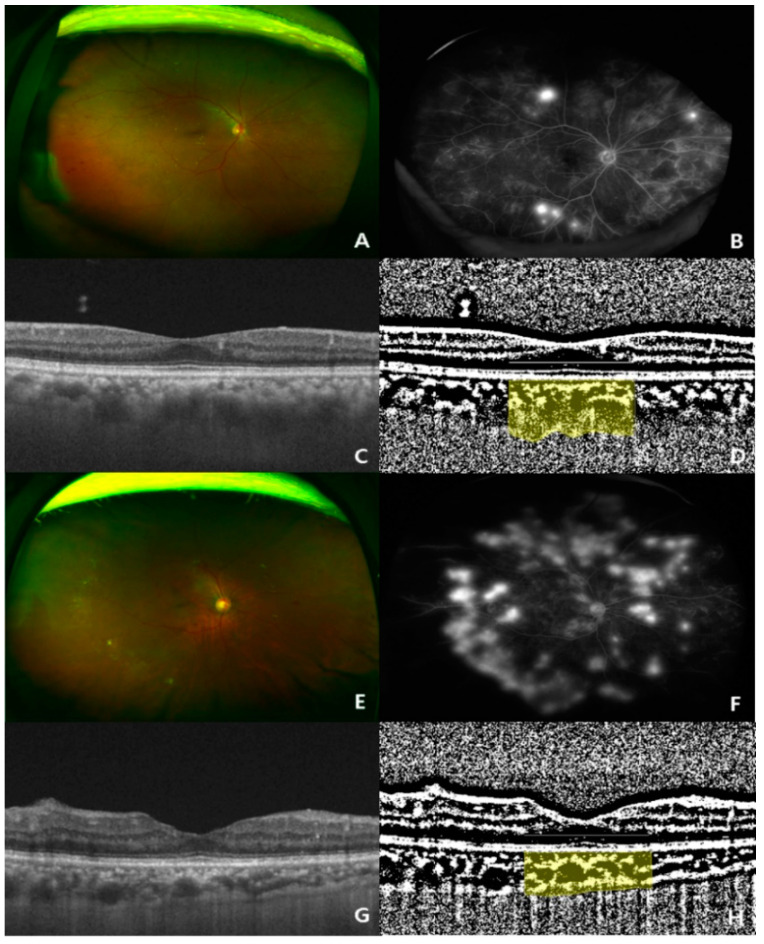
Two representative cases (ultra-wide-fundus photo, ultra-wide-fluorescein angiography, optical coherence tomography, and binary image of optical coherence tomography) showing choroidal thickening and thinning in treatment-naïve eyes with proliferative diabetic retinopathy (PDR). (**A**–**D**) Representative case of a 73-year-old woman with a high choroidal vascularity index (CVI, 0.723%; subfoveal choroidal thickness, 342 μm) in the eyes; (**E**–**H**) representative case of a 50-year-old man with a low CVI index (CVI, 0.653%; subfoveal choroidal thickness, 214 μm) in the eyes.

Horizontal and vertical OCT B-scan images passing at the fovea center from the five-line cross-scan were selected. Image processing in OCT B-scans was performed using the method described by Agrawal et al. [11]. The binarization of the OCT B-scans was performed using the ImageJ software (http://imagej.nih.gov/ij, accessed on 20 August 2021, version 1.80). Two representative cases for binarization and segmentation to obtain CVI are shown in Figure 1. The total choroid area with a width of 1500 μm centered on the fovea was selected using the polygon tool and added to the ROI manager. The image was converted to an 8-bit image and adjusted using the Niblack autolocal threshold method. The dark pixels were selected using the color threshold tool and this area was also added to the ROI manager as a luminal area. The area of white pixels was defined as the stromal area. CVI was defined as the luminal area/total choroid area.

### 2.6. Statistical Analyses

Two independent observers (J.T.K. and I.G.L.) blinded to the clinical data of the patients measured the SFChT and CVI, and the mean values were used for the statistical analysis. The data are presented as the mean ± standard deviation. Statistical analyses were performed using the SPSS software (version 25.0; IBM Corp., Armonk, NY, USA). One-way analysis of variance (ANOVA) with post hoc analysis using Tukey’s method was used to compare several variables, including age, BCVA, IOP, CVI, SFChT, and CRT, between the DR groups. Additionally, the analysis of covariance (ANCOVA) was used to determine the age-adjusted changes in SFChT and CVI. Statistical significance was defined as a *p*-value < 0.05.

The following four ocular parameters from one eye (right eye) of each patient were recorded and analyzed to investigate the correlation with CVI: BCVA, refractive error (spherical equivalent), SFChT, and CRT. The following 14 systemic profiles were recorded to analyze the correlation between CVI and the parameters obtained from the systemic work-up: age, systolic BP, diastolic BP, BMI, height, weight, blood urea nitrogen, creatinine, blood urea nitrogen/creatinine ratio, estimated glomerular filtration rate, serum levels of calcium and phosphorus, urinary microalbumin level, and urinary microalbumin/creatinine ratio.

## 3. Results

### 3.1. Baseline Characteristics

A total of 131 patients with type 2 diabetes (85 men and 76 women) and treatment-naïve DR eyes were enrolled in this study. The mean age of the patients was 55.9 ± 12.6 years, and the mean duration of DM was 12.8 ± 16.4 years. The eyes were categorized according to the DR severity: no DR (32 eyes from 32 patients), mild-to-moderate NPDR (33 eyes from 33 patients), severe NPDR (34 eyes from 34 patients), and naïve PDR (32 eyes from 32 patients). Thirty eyes from 30 age-matched subjects were included as the control group.

No significant differences were observed in age, DM duration, systolic BP, BMI, or IOP between the groups. However, the HbA1c levels, diastolic BP, BCVA, refractive error, CRT, SFChT, and CVI were significantly different between the groups. The serum HbA1c level and diastolic BP were greater in patients with naïve PDR than in those with other severities of DR. The baseline characteristics of the patients are summarized in Table 1.

### 3.2. Changes in CVI and SFChT in Eyes with DR

**Figure 2:**
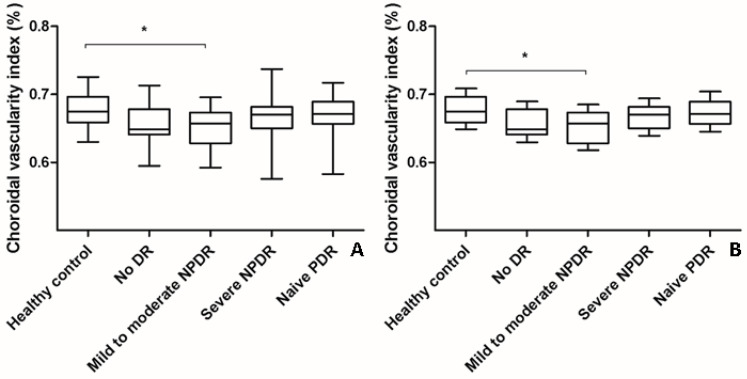
Changes in choroidal vascularity index (CVI) according to the severity of diabetic retinopathy (DR). (**A**) The mean CVI of eyes with mild-to-moderate NPDR was significantly lower than that of healthy control eyes (analysis of variance, post hoc * *p* = 0.045). The mean CVI of eyes with treatment-naïve PDR and severe NPDR were higher than that of eyes with mild-to-moderate NPDR and no DR. However, the changes were not significant as indicated by a post hoc analysis. Data are shown as mean ± standard deviation. (**B**) Age-adjusted changes in CVI were similar to the changes in CVI without adjustment (analysis of covariance, post hoc, * *p* = 0.042). Data are shown as mean ± standard error. CVI; choroidal vascularity index; DR, diabetic retinopathy; NPDR, non-proliferative diabetic retinopathy; PDR, proliferative diabetic retinopathy.

The mean CVI of eyes with naïve PDR (0.668% ± 0.029%) or severe NPDR (0.666% ± 0.032%) was higher than that of eyes with mild-to-moderate NPDR (0.652% ± 0.032%) or no DR (0.658% ± 0.031%) (ANOVA, post-hoc). However, the changes were not significant even after adjustments were made for age (ANCOVA, post-hoc) (Table 1, Figure 2). The mean CVI of eyes with mild-to-moderate NPDR was significantly lower than that of healthy control eyes (Table 1, Figure 2).

The changes were also significant after adjustments were made for age (ANCOVA, post hoc). The SFChT in eyes with naïve PDR (319.7 ± 88.5 µm) was significantly greater than that in eyes with mild-to-moderate NPDR (269.1 ± 80.8 µm) and healthy control eyes (274.3 ± 31.9 µm) (ANOVA, post hoc, *p* = 0.04; *p* = 0.003, respectively). The changes in SFChT showed a similar pattern after adjustments were made for age. The CRT in eyes with naïve PDR (252.7 ± 51.5 μm) was greater than that in healthy control eyes (221.0 ± 22.3 μm) (*p* = 0.043). The inter-observer reproducibility of CVI and SFChT ranged from 0.920 to 0.940 and 0.986 to 0.990, respectively.

**Figure 3:**
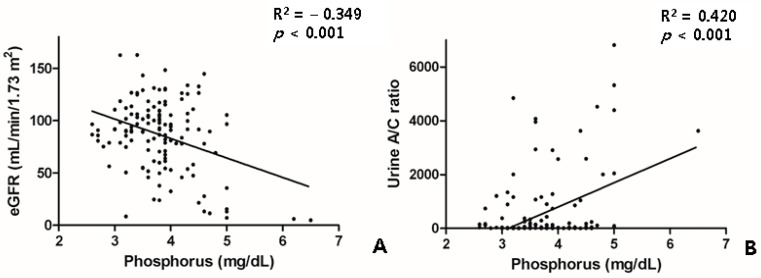
Scatter plot showing relationship between the phosphorus level and the renal profiles of eGFR (**A**) and the urine A/C ratio (**B**). A/C, albumin/creatinine; eGFR, estimated glomerular filtration rate.

The patients with treatment-naïve PDR or severe NPDR had higher levels of blood urea nitrogen, creatinine, estimated glomerular filtration rate (eGFR), and urine albumin, as well as a higher urine albumin/creatinine ratio. The changes in the renal parameters of the patients with DR are shown in Table 2. The phosphorus levels were not different between the DR groups. However, the phosphorus levels were closely correlated with the eGFR and the urine A/C ratio (Figure 3).

### 3.3. Association between CVI and Ocular and Systemic Factors Related with Diabetes

**Figure 4:**
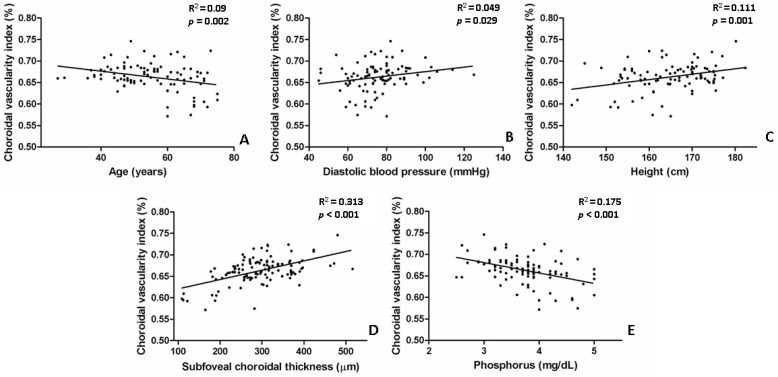
Scatter plot between choroidal vascularity index (CVI) and several parameters that showed statistical significance in univariate linear regression. (**A**,**E**) Age and serum levels of phosphorus were negatively correlated with CVI. (**B**–**D**) Subfoveal choroidal thickness, body height, and diastolic blood pressure were positively correlated with CVI.

In the univariate analysis, lower CVI values were significantly associated with the following parameters: older age (*p* = 0.002), lower diastolic BP (*p* = 0.029), lower body height (*p* = 0.001), lower SFChT (*p* < 0.001), and higher serum levels of phosphorus (*p* < 0.001) (Table 3, Figure 4). In the multivariate analysis, lower CVI values were associated with older age (*p* = 0.013), lower SFChT (*p* < 0.001), and higher serum levels of phosphorus (*p* = 0.002) (Table 3). Two representative cases of higher and lower CVIs of eyes with naïve PDR among patients with early-stage and late-stage CKD are shown in Figure 1.

## 4. Discussion

In this study, changes in the CVI were systematically investigated in patients with diabetes using the image binarization of SS-OCT scans. The primary finding of the current study is that the choroidal vasculature is associated with serum phosphorus levels.

Sonoda et al. have previously described a quantification method for the luminal area in the choroid (defined as CVI) using the Niblack binarization conversion image of an OCT B-scan [24]. Several studies have been performed to investigate the changes in CVI in various diseases, including central serous chorioretinopathy [11], Vogt–Koyanagi-Harada disease [25], and age-related macular degeneration [26]. CVI has a lower variability and is influenced by fewer physiologic factors [23]. Nevertheless, the present study revealed that the CVI of patients with diabetes is affected by several factors.

First, the serum levels of phosphorus were found to be negatively correlated with CVI. Phosphorus is an essential component of the cell membranes and nucleic acids [27]. Dietary phosphorus is normally absorbed in the small intestine, and excess phosphorus is excreted and reabsorbed by the kidneys [28]. The homeostasis of phosphorus is regulated by complex interactions among the intestines, parathyroid glands, kidneys, and bones under the regulatory action of the parathyroid hormone–vitamin D axis and fibroblast growth factor-23 (FGF-23) [29]. Among these regulatory organs, the kidneys play a major role in phosphorus homeostasis [29]. The progression of CKD leads to hyperphosphatemia through the diminished infiltration and excretion of phosphorus, resulting in the progression of secondary hyperparathyroidism [30,31,32]. Moreover, hyperphosphatemia is related to bone fragility and a high bone turnover, leading to vascular calcification and cardiovascular disease. Therefore, in diabetic patients with CKD, hyperphosphatemia is considered a risk factor and an important prognostic marker of cardiovascular morbidity and mortality [31,33]. Furthermore, even in patients without CKD, a higher level of serum phosphorus that is still within the normal range is significantly associated with myocardial infarction and coronary death [34]. Therefore, decreased CVI in eyes with DR might be associated with the vascular calcification of choroidal vasculature or the cardiovascular system.

Second, SFChT was found to be positively correlated with CVI. Several previous studies have described a correlation between CVI and SFChT. Agrawal et al. reported that a higher CVI was associated with a thicker SFChT in a normal population [23]. Kim et al. also described that a thicker subfoveal choroid was significantly associated with a higher CVI [15]. Our results are in good agreement with those of previous reports. The consistency reflects the validity of CVI in this study. Thus, the factors that correlated with SFChT could potentially be associated with changes in CVI.

Third, age was found to be negatively correlated with the CVI in the univariate and multivariate regression analyses. A correlation between age and choroidal thickness has been described previously [35,36]. Choroidal thickness diminishes with age, and the CVI could be potentially affected by SFChT. Agrawal et al. reported that age and CVI were correlated in a linear regression analysis [23]. These results are consistent with the findings of the current study.

Finally, in the present study, diastolic BP and body height were found to be associated with the CVI in a univariate regression analysis, although the multivariate analysis did not show any statistical significances. These factors might potentially be associated with the choroidal vasculature according to the distribution of systemic parameters in the enrolled subjects. The correlation between diastolic BP and CVI was previously reported in a univariate linear regression, despite showing no significance being seen in a multivariate analysis [23]. This point is similar to the findings of the current study.

The SFChT changes in eyes with DR or diabetic eyes without DR have been intensely investigated by several studies [15,37,38,39,40,41,42,43,44,45]. However, the relationship between DR severity and SFChT is controversial. Some studies have reported that SFChT in eyes with DR increases as DR progresses [38,39,40], while others have suggested that SFChT decreases in patients with diabetes [15,41,42,43,44]. Recently, Wang et al. reported an increase in SFChT in the early stage of DR, but this decreased with DR progression [45]. Moreover, Endo et al. showed that the SFChT of eyes with severe NPDR was thicker than that of normal controls only in the DM treatment group [37]. Changes in choroidal vasculature have also been seen in patients with diabetes [10], and studies have demonstrated that CVI is significantly decreased with a worsened DR [10,14,15]. This study also showed that the CVI of eyes with mild-to-moderate NPDR was lower than that of healthy control eyes. However, this study did not show a similar decreasing trend in CVI as DR progresses. Considering the close correlation between CVI and SFChT, we think that these inconsistencies might be related to the conflicting findings of SFChT in eyes with DR or diabetic eyes without DR. We think that the variability in both SFChT and CVI suggests the presence of several confounding factors. Thus, we attempted to find the relationship between CVI and several systemic profiles.

Finally, we discovered that the decrease in CVI in patients with diabetes was associated with phosphorus retention. Thus, the correlation between CVI and a few factors suggests that relatively healthy and young patients without phosphorus retention have a higher CVI than unhealthy and older patients with phosphorus retention.

Previously, we investigated the correlation between SFChT and systemic profiles [21,22,46]. These studies suggested a correlation between choroid and CKD. However, CVI is known to be less variable than SFChT and is affected by fewer parameters [23]. Thus, in this study we tried to investigate the correlation between CVI and renal profiles. We showed that CVI was less affected by key renal profiles, including eGFR and the urine albumin/creatinine ratio. We think that the lack of correlation also suggests a lower variability of CVI.

This study has several limitations. First, this was a retrospective study in which several variables such as diurnal variation could be confounding factors. Nevertheless, only patients with treatment-naïve DR eyes were included in order to reduce several confounding factors, because treatments such as panretinal photocoagulation, focal laser, and intravitreal injection can induce choroid thinning [47,48,49]. Second, the effects of systemic medications were not revealed. All the enrolled patients were prescribed medications to help control their systemic disease and these medications might have affected their vasculature, although the actual effect is not known. Third, only a central scan of 1500 μm on the fovea was analyzed in this study. Future analyses of larger areas may provide additional information. However, the choroidal vasculature of a single foveal scan could be used to represent the entire choroidal vasculature [50]. Finally, drawing a conclusion would be premature based on these retrospective uncontrolled data. A well-controlled prospective study might lead to a robust conclusion.

In summary, we showed that CVI was negatively correlated with age and the serum level of phosphorus and positively correlated with SFChT. The elucidation of correlations between CVI and systemic profiles in patients with DR might provide insight into the physiology of the choroidal vasculature.

## Figures and Tables

**Table 1:** Baseline characteristics of patients.

Variable	Healthy Controls	No DR	Mild/Moderate NPDR	Severe NPDR	Treatment-Naïve PDR	*p*-Value ^†^
Number of patients	30	32	33	34	32	-
Number of eyes	30	32	33	34	32	-
Age (years)	59.1 ± 13.9	58.2 ± 16.5	58.7 ± 11.0	55.9 ± 9.3	49.6 ± 9.9	0.058
DM Duration (years)	-	8.5 ± 5.2	14.0 ± 9.3	18.9 ± 26.1	12.0 ± 7.5	0.11
Hemoglobin A1c, %	-	9.1 ± 2.1	7.9 ± 0.6	7.7 ± 1.4	8.8 ± 2.3	0.032
Number of Patients with Hypertension, *n* (%)	-	20 (62.5%)	25 (86.2%)	27 (79.4%)	24 (82.7%)	-
Systolic BP (mmHg)	-	123.2 ± 13.5	126.3 ± 13.5	131.2 ± 20.1	132.3 ± 19.1	0.194
Diastolic BP (mmHg)	-	70.2 ± 7.2	71.2 ± 8.5	75.7 ± 12.0	82.4 ± 18.1	0.006
BMI (kg/m^2^)	25.2 ± 2.8	24.8 ± 4.5	26.1 ± 3.9	25.2 ± 3.8	25.8 ± 4.6	0.347
Height	1.60 ± 0.07	1.62 ± 0.08	1.63 ± 0.08	1.65 ± 0.08	1.67 ± 0.08	0.09
Weight	64.6 ± 9.1	64.1 ± 10.8	68.9 ± 10.1	68.1 ± 13.5	74.2 ± 15.6	0.191
BCVA (LogMAR)	0.06 ± 0.08	0.1 ± 0.07	0.06 ± 0.08	0.12 ± 0.27	0.24 ± 0.32	0.027
IOP (mmHg)	15.9 ± 3.0	15.6 ± 5.2	16.7 ± 3.6	16.2 ± 3.2	16.1 ± 2.8	0.888
SE (diopter)	−0.42 ± 1.82	0.51 ± 0.41	−0.56 ± 1.61	−0.62 ± 1.83	−1.38 ± 1.98	0.042
CRT (µm)	221.0 ± 22.3	231.4 23.6	245.6 26.9	244.8 62.4	252.7 ± 51.5	0.043
SFChT (µm)	274.3 ± 31.9	256.8 ± 16.1	269.1 ± 80.8	305.4 ± 72.2	319.7 ± 88.5	0.002
SFChT (µm), age adjusted *	272.6 ± 11.4	250.2 ± 15.7	263.5 ± 12.4	299.9 ± 10.5	299.5 ± 9.7	0.002
CVI	0.677 ± 0.023	0.658 ± 0.031	0.652 ± 0.032	0.666 ± 0.032	0.668 ± 0.029	0.034
CVI, age adjusted *	0.678 ± 0.007	0.658 ± 0.007	0.654 ± 0.005	0.667 ± 0.005	0.666 ± 0.004	0.045

Data are presented as number (%) or mean ± SD, unless otherwise indicated; * data are presented as mean ± standard error. ^†^ Analysis of variance among the groups. BCVA, best-corrected visual acuity; BP, blood pressure; BMI, body mass index; CRT, central retinal thickness; CVI, choroidal vascularity index; DM, diabetes mellitus, DR, diabetic retinopathy; HbA1c, hemoglobin A1c; IOP, intraocular pressure; NPDR, non-proliferative diabetic retinopathy; PDR, proliferative diabetic retinopathy; SE, spherical equivalent; SFChT, subfoveal choroidal thickness.

**Table 2:** Comparison of renal profiles of patients with diabetic retinopathy.

Variable	No DR	Mild/Moderate NPDR	Severe NPDR	Treatment-Naïve PDR	*p*-Value ^†^
BUN (mg/dL)	13.88 ± 5.33	17.29 ± 5.18	18.10 ± 9.17	22.26 ± 13.26	0.012
Creatinine (mg/dL)	0.76 ± 0.21	0.80 ± 0.24	0.96 ± 0.49	1.72 ± 20.5	0.003
BUN/Cr ratio	18.69 ± 7.01	21.99 ± 6.09	19.93 ± 7.52	17.81 ± 7.09	0.072
eGFR (mL/min/1.73 m^2^)	99.14 ± 22.23	97.11 ± 21.82	88.52 ± 30.22	73.73 ± 41.51	0.004
Phosphorus (mg/dL)	3.67 ± 0.47	3.76 ± 0.65	3.72 ± 0.47	3.94 ± 0.76	0.286
Calcium (mg/dL)	9.19 ± 0.38	9.03 ± 0.35	9.05 ± 0.36	8.94 ± 0.51	0.197
Urine albumin (mg/L)	41.86 ± 105.43	146.16 ± 319.31	540.54 ± 1911.99	1399.0 ± 2299.73	0.004
Urine A/C ratio	26.23 ± 67.08	163.06 ± 482.49	291.88 ± 658.22	1387.98 ± 1958.73	<0.001

Data are presented as number (%) or mean ± SD unless otherwise indicated; A/C, albumin/creatinine; BUN, blood urea nitrogen; Cr, creatinine; DR, diabetic retinopathy; eGFR; estimated glomerular filtration rate; NPDR, non-proliferative diabetic retinopathy; PDR, proliferative retinopathy. ^†^ Analysis of variance among the groups.

**Table 3:** Univariate and multivariate regression analyses of the relationship between choroidal vascularity index and systemic and ocular parameters.

Variables	Univariate Linear Regression			Multivariate (Stepwise) Linear Regression	
	Standardized β	R^2^	*p*-Value	Standardized β	*p*-Value
Age (years)	−0.312	0.09	0.002 *	−0.207	0.013 *
Systolic BP (mmHg)	0.115	0.013	0.263		
Diastolic BP (mmHg)	0.222	0.049	0.029 *	0.058	0.454
BMI (kg/m^2^)	−0.013	0.000	0.897		
Height (m)	0.333	0.111	0.001 *		
Weight (kg)	0.031	0.031	0.086		
BCVA(LogMAR)	−0.086	0.007	0.404		
SFChT (µm)	0.560	0.313	<0.001 *	0.473	<0.001 *
CRT (µm)	−0.064	0.004	0.534		
Spherical equivalent (diopter)	−0.051	0.003	0.621		
BUN (mg/dL)	−0.053	0.003	0.606		
Cr (mg/dL)	−0.024	0.001	0.819		
BUN/Cr	−0.031	0.001	0.766		
eGFR (mL/min/1.73 m^2^)	0.105	0.011	0.305		
Phosphorus (mg/dL)	−0.422	0.175	<0.001 *	−0.269	0.002 *
Calcium (mg/dL)	−0.053	0.002	0.467		
Urine albumin (mg/L)	0.108	0.006	0.137		
Urine A/C ratio	0.068	0.005	0.354		

Multivariate R^2^ = 0.399; * *p*<0.01 A/C, albumin/creatinine; BCVA, best-corrected visual acuity; BMI, body mass index; BP, blood pressure; BUN, blood urea nitrogen; Cr, creatinine; CRT, central retinal thickness; eGFR, estimated glomerular filtration rate.

## Data Availability

The datasets during and/or analyzed during the current study are available from the corresponding author on reasonable request.
